# Research on the Impact Initiation Behavior of PTFE/Al/Ni_2_O_3_ Reactive Materials

**DOI:** 10.3390/polym14214629

**Published:** 2022-10-31

**Authors:** Can Liu, Yi-Yang Dong, Yu-Yang Fan, Yi Yang, Jing-Yun Zhao, Ke Wang, Xiao-Jun Liu

**Affiliations:** 1 Institute for Advanced Study, Nanchang University, Nanchang 330031, China; 2 Institute of Aeronautics and Astronautics, Nanchang University, Nanchang 330031, China

**Keywords:** PTFE/Al/Ni_2_O_3_, reactive material, drop hammer test, quasi-static compressive properties, impact initiation behavior

## Abstract

PTFE/Al reactive material is an energetic material that releases energy under impact conditions, resulting in a wide range of application prospects. In order to improve its damage ability—considering the higher heat of the reaction per unit mass when Ni_2_O_3_ is involved in the aluminothermic reaction—we designed and studied PTFE/Al/Ni_2_O_3_, a reaction material based on polytetrafluoroethylene (PTFE). We also designed two other kinds (PTFE/Al, PTFE/Al/CuO) for comparative study, with the mass fraction of the metal oxides added at 10%, 20%, and 30%, respectively. The quasi-static compression properties and impact initiation behavior of the material were investigated by a universal material testing machine and a drop hammer test. The reaction process of different materials under a high strain rate was recorded using a high-speed camera. The results show that with the increase in Ni_2_O_3_ content, the strength of the PTFE/Al/Ni_2_O_3_ reactive material shows an increasing trend followed by a decreasing trend. Among the three reactive materials, when the content of Al/Ni_2_O_3_ reaches 30 wt.%, the reaction duration is the longest (at 4 ms) and the reaction fireball is the largest. The addition of Ni_2_O_3_ is helpful to improve the reactivity and reaction duration of the PTEF/Al reactive material.

## 1. Introduction

Reactive material is a new type of energetic material. It was first discovered in the 1970s and then began to be widely studied. Unlike general energetic materials, reactive materials can remain stable under normal conditions. The main types of reactive materials are thermite (such as Al/CuO), metal-polymer mixtures (such as Al/PTFE), intermetallic compounds (such as Al/Ni), metastable intermolecular compounds (such as Al/MoO_3_), and so on [1,2,3,4]. The energy excitation of reactive materials requires an external force to produce plastic deformation or breakage at a high strain rate, while it is difficult to stimulate the reaction with general excitation methods, such as combustion and electric shock [5]. At present, the most widely studied PTFE-based reactive material is PTFE/Al (73.5 wt.%/26.5 wt.%) [6,7] in intermetallics. Its unit mass energy and unit volume energy can reach 3.5 times and 5 times that of trinitrotoluene (TNT) explosives [8]. Under high-speed impact conditions, PTFE/Al undergoes intense combustion and detonation reactions, so it can be used in kinetic energy damage units.

Among all PTFE-based reactive materials, many scholars have carried out a large number of mechanical properties tests and energy release characteristics studies [9]. Since the 1970s, Mock et al. [10] found that fire light would occur when PTFE/Al impacted the target plate at high speeds. Joshi et al. [11] proposed the pressing and sintering process of PTFE/Al energetic reactive materials. Although the preparation of PTFE/Al reactive materials was convenient and safe, the strength and reactivity of PTFE/Al reactive materials were weak. Cai et al. [12,13] carried out a large number of mechanical properties tests on PTFE/Al and PTFE/Al/W reactive materials, including a quasi-static compression test, drop hammer impact test, and dynamic split Hopkinson pressure bar (SHPB) test. Their results showed that the addition of W can enhance performance in the strength of reactive materials, but it has no significant effect on improving its reactivity. There are already many studies on the mechanical properties of different kinds of polymers in academia. Among them, Amjadi et al. [14] realized different deformation ratios by applying a compression process to high-density polyethylene materials and carried out tensile experiments on the samples. It was found that with the increase in the deformation rate, the tensile strength was different. Zhou and Ran et al. [15,16] studied the impact properties and mechanical properties of micro- and nano-scale PTFE/Al/CuO reactive materials. They found that the addition of an Al/CuO aluminothermic agent can improve the reaction rate and strength of the material, but the addition of nano-scale PTFE/Al results in a material strength that is too low and difficult to form. The reactivity and reaction mechanisms of PTFE/Al reactive materials have also been extensively investigated. Tao et al. [17] used reactive molecular dynamics simulation methods and density functional theory to predict and calculate the reaction mechanism of PTFE/Al reactive materials; they studied the reaction products and reaction processes through experiments. Therefore, in the present study, we consider improving the reactivity of the reaction material by adding metal oxides to the reaction material to thermally react with Al.

We consider adding Al/Ni_2_O_3_ aluminum thermite to PTFE/Al. The reaction heat of Al/Ni_2_O_3_ thermite is high [18], and its reaction heat can reach 5229 cal/cm^3^. Considering the phase change factor, the adiabatic reaction temperature of Al/Ni_2_O_3_ thermite can reach 3187 °C, and the sensitivity of Al/Ni_2_O_3_ thermite is also very high. These factors are very beneficial for promoting the occurrence and diffusion of the reaction and improving the reaction efficiency.

In this work, we prepared three kinds of reactive materials, namely PTFE/Al/Ni_2_O_3_, PTFE/Al/CuO, and PTFE/Al. We mainly studied the effect of the addition of Ni_2_O_3_ on the mechanical properties and reactivity of PTFE/Al reactive materials under different addition ratios. Further, we used high-speed cameras with higher frame rates and specially designed hammer heads to better observe the entire process of specimen deformation, failure, and ignition.

## 2. Materials and Methods

In this experiment, three kinds of reactive materials—PTFE/Al, PTFE/Al/CuO, and PTFE/Al/Ni_2_O_3_—were prepared using the molding sintering method. The average sizes of the PTFE, Al, CuO, and Ni_2_O_3_ particles used in the experiment were 30 μm, 1 μm, 50 nm, and 50 nm, respectively. The reaction equations of PTFE and Al under vacuum conditions, and the aluminothermic reaction equations of Al with CuO and Ni_2_O_3_, respectively, are as follows:

4Al + 3C_2_F_4_ = 4AlF_3_ + 6C (1)

2Al + 3CuO = Al_2_O_3_ + 3Cu (2)

2Al + Ni_2_O_3_ = Al_2_O_3_ + 2Ni (3)

According to the reaction equation, the zero-oxygen balance reaction mass ratio of PTFE and Al is 73.5:26.5, the reaction mass ratio of CuO and Al is 81.5:18.5, and the reaction mass ratio of Ni_2_O_3_ and Al is 75.4:24.6. Based on the mentioned reaction ratios, a series of material formulations were designed as follows.

The procedure is as follows: Prepare the powdered reactive materials in beakers according to the proportions in Table 1, and add 150 mL of anhydrous ethanol. Grind the materials using the ball mill adjusted to 250 r/min, and stir them for 30 min. Then, evaporate them using a rotary evaporator for 2 h, and dry the materials in a vacuum oven at 60 °C for 24 h. Press the generated powder into a cylinder of ϕ 10 mm × 60 mm using a mold and a hydraulic press. Stand the material for 16 h to eliminate residual stress, and sinter the materials in a vacuum sintering furnace.

**Figure 1:**
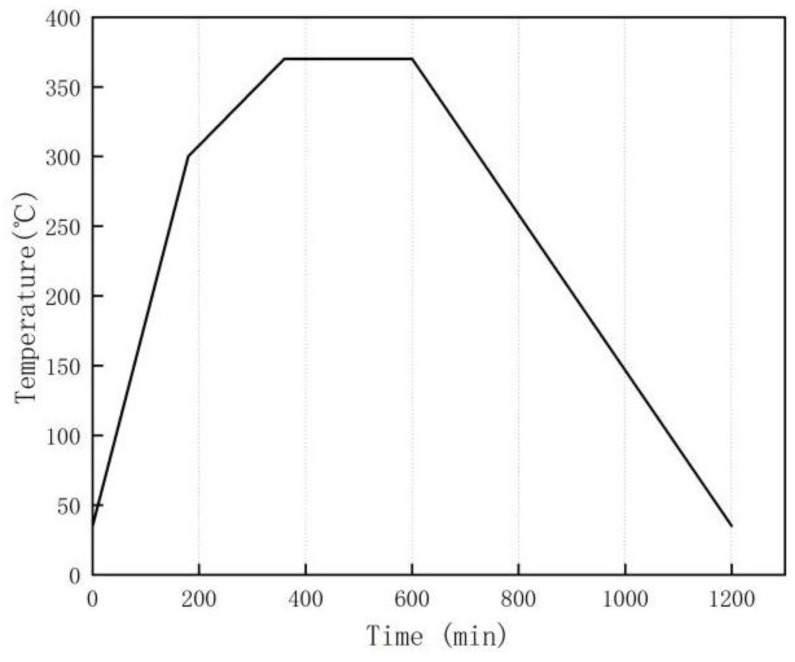
Sintering process.

Previous research [16,19] indicates that the melting temperature of PTFE is 327 °C, and it would decompose when the temperature exceeds 400 °C. Therefore, the best sintering temperature is considered to be 370 °C. The sintering process is to heat for 3 h to raise the temperature of the furnace chamber from room temperature to 300 °C. Then, over 3 h, increase the temperature from 300 °C to 370 °C. Keep the temperature at 370 °C for 4 h, and then let the chamber naturally cool to room temperature. The sintering process is shown in Figure 1.

**Figure 2:**
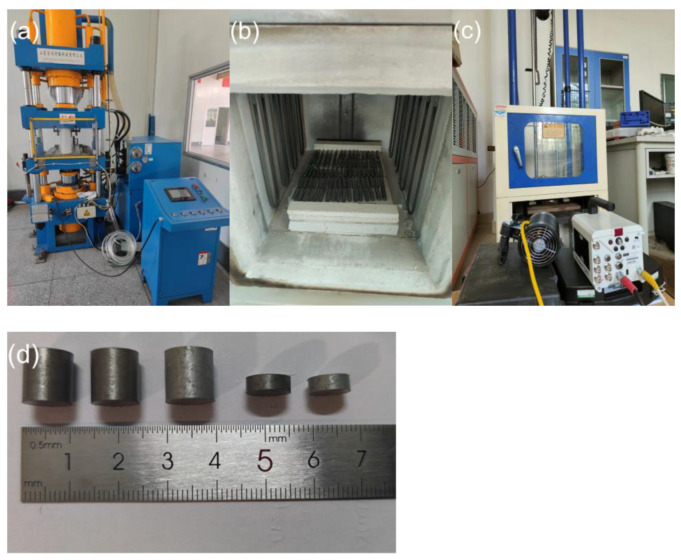
(**a**) Hydraulic press; (**b**) Vacuum sintering furnace; (**c**) Drop hammer impact test machine; (**d**) Sintered samples.

The sintered material is processed into ϕ 10 mm × 10 mm and ϕ 10 mm × 3 mm cylinders using a CNC lathe, as shown in Figure 2, and then used in quasi-static compression and dynamic impact experiments, respectively. The microstructure of the material was observed using a scanning electron microscope (SEM), and the phase composition of the raw material and the reaction product was examined using an X-ray diffractometer (XRD). We carried out the quasi-static compression test 3 times on each material using a universal testing machine. According to the experimental conditions of GB/T1039-1992, the indenter travels at a rate of 0.01 mm/s and a strain rate of 0.001 s^−1^ under standard laboratory conditions. We carried out the dynamic impact test 5 times on each material using a drop hammer impact tester. The temperature was between 15~30 °C, the relative humidity was not more than 80%, and the sample was kept dry. The weight of the drop hammer was 7.4 kg, the height of the drop hammer was 95 cm, and the whole process of sample deformation, failure, and ignition was recorded using a high-speed camera at a frame rate of 63,000 fps.

## 3. Results

### 3.1. The Characteristics of the Materials

**Figure 3:**
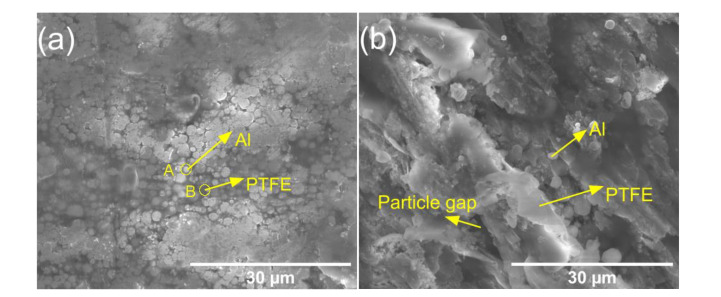

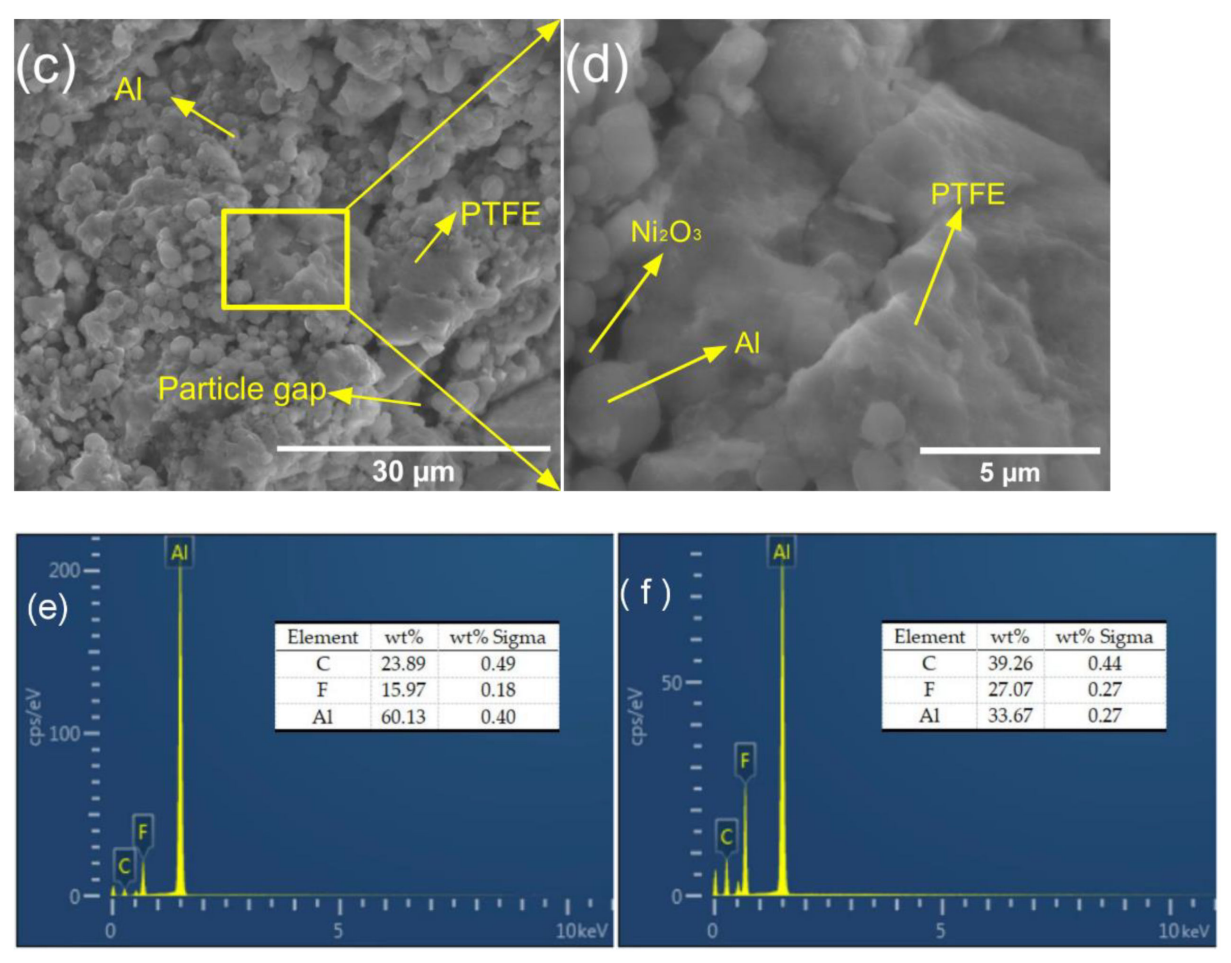
Microstructure of different reactive materials after sintering. (**a**) PTFE/Al; (**b**) PTFE/Al/CuO; (**c**) PTFE/Al/Ni_2_O_3_; (**d**) Local magnification view of (**c**); (**e**) SEM at A point of (**a**); (**f**) SEM at B point of (**a**).

The surface morphologies of the PTFE/Al, PTFE/Al/CuO, and PTFE/Al/Ni_2_O_3_ reactive materials were observed through SEM, as shown in Figure 3. It can be seen that the sintered PTFE was not fibrous but aggregated together, where some large spherical Al particles were encapsulated in the PTFE substrate, and the particles were distributed evenly. The energy-dispersive spectrometer (EDS) analysis results of points A and B are shown in Figure 3e,f, which further supports our judgment. It can be seen from Figure 3b,c that, as the PTFE content decreased, the effect of the encapsulating particles was weakened, the combination with Al particles became loose, and a small amount of gap is visible, locally. The CuO and Ni_2_O_3_ particles are difficult to observe at the current magnification because of their small size. After the local enlargement of Figure 3c, as shown in Figure 3d, it can be seen that Al and Ni_2_O_3_ particles are covered in PTFE.

### 3.2. Quasi-Static Compressive Mechanical Properties

**Figure 4:**
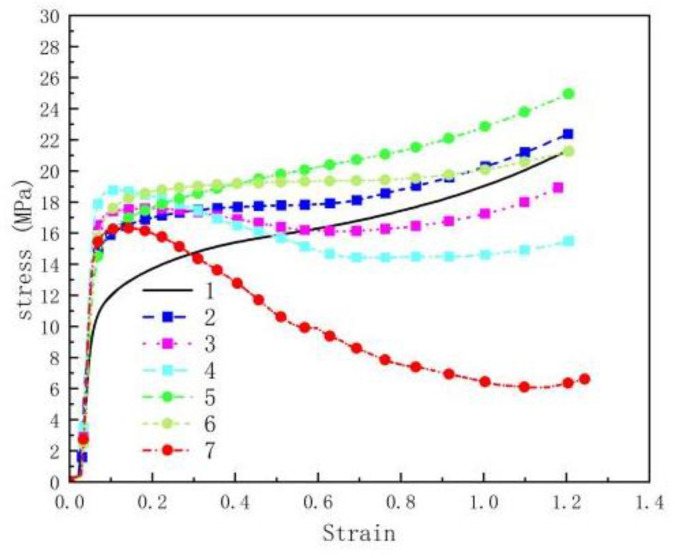
Quasi-static compressive stress-train curves of different reactive materials.

**Figure 5:**
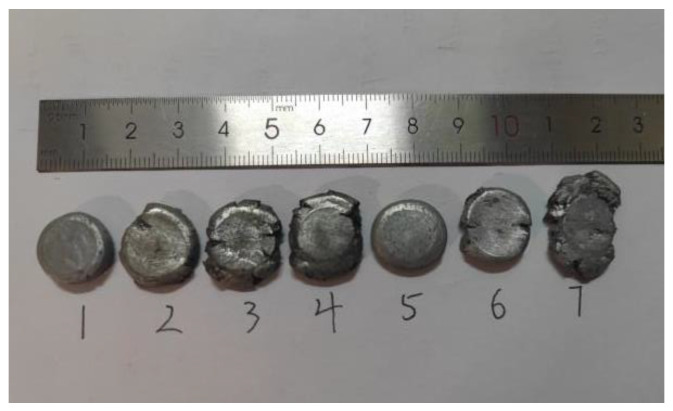
Samples after quasi-static compression.

According to a large amount of research literature [15], the comprehensive mechanical properties of sintered reactive materials are better than those of unsintered reactive materials. Therefore, this work only studies the mechanical properties of sintering reactive materials. The quasi-static compressive mechanical properties of all the reactive material samples were tested 3 times. After averaging the data, we drew the force and displacement images. After further data processing, the calculated real stress–strain curves are shown in Figure 4. In addition, images of the seven samples after the quasi-static compression tests are shown in Figure 5.

It can be seen from Figure 4 that the elastic stage exists in all seven groups of reactive materials. Among them, the strength of the reactive materials added with CuO and Ni_2_O_3_ was higher than that of PTFE/Al. These results indicate that the addition of CuO and Ni_2_O_3_ particles in the PTFE/Al material can effectively transfer the stress between the matrix and the particles, thus improving the strength of the material. Among them, the elastic-plastic stages of No.1# to No.6# reactive materials are more obvious than No.7#, which fails quickly after its elastic stage. To explain this phenomenon, the volume fraction of oxides in each reactive material is shown in Table 2.

From the table, it can be seen that the volume fraction of Ni_2_O_3_ is higher than that of CuO in the same ratios of PTFE/Al/Ni_2_O_3_ and PTFE/Al/CuO reactive materials, and the oxide volume fraction of sample No.7# is up to 4.69%. Therefore, the reason why there is no elastic-plastic stage for reactive material No.7# is that the PTFE content in the material is too small, leading the particle gap to be too large and the reinforcing phase particles (Al and Ni_2_O_3_) being unable to be coated completely and uniformly by the PTFE. At the same time, too many Al and Ni_2_O_3_ particles will also destroy the continuity of the PTFE matrix, and it will soon fail after the elastic stage. For the No.5# and No.6# reactive materials, when the volume fraction of the oxide is low, the increase in the additive content will not significantly damage the continuity of the PTFE matrix. Zhou et al. [20] found that when the content of Al/CuO increases from 25 wt.% to 50 wt.%, which is equal to when the volume fraction increases from 3.23% to 6.45%, the reactive material did not exhibit a distinct elastic-plastic stage and performed poorly in terms of strength. When the volume fraction of CuO increases from 1.61% to 3.23%, the stress-strain curves of the reactive materials are similar. This indicates that a similar situation will occur if the content of CuO continues to increase in this experiment.

**Figure 6:**
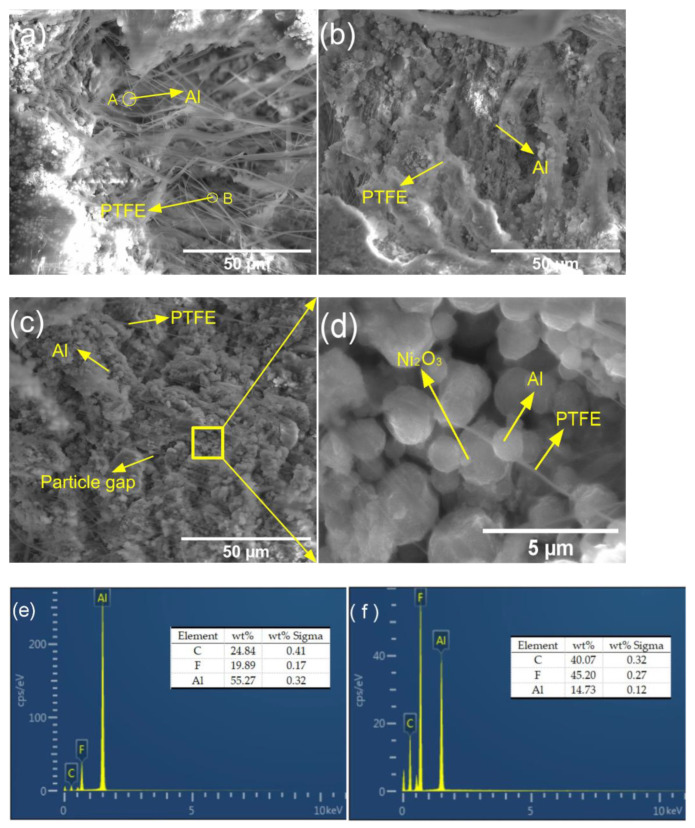
The fracture morphology of different reactive materials after quasi-static compression. (**a**) PTFE/Al; (**b**) PTFE/Al/CuO; (**c**) PTFE/Al/Ni_2_O_3_; (**d**) Local magnification view of (**c**); (**e**) SEM at A point of (**a**); (**f**) SEM at B point of (**a**).

Also, we observed the cracks in the reactive materials of No.1#, No.4#, and No.7# after quasi-static compression through scanning electron microscopy, as shown in Figure 6. As can be seen from Figure 6a, the PTFE content of reactive material No.1# was high, and a large amount of fibrous PTFE was present at the crack, with a diameter of about 0.5 μm. The PTFE particles broke under the shear and extension of the surrounding polymer melt during the compounding process, and highly oriented PTFE fibers were formed when sheared on the hot plane surface [21,22,23]. Therefore, under the actions of shear force and elongation force, the fiber structure with a high aspect ratio was formed, and the distribution of reinforced phase particles (Al) at the crack was smaller—only scattered in the fibrous PTFE. SEM analysis of the fibers and fiber-wrapped particles at points A and B, as shown in Figure 6e,f, further confirmed our judgment. Figure 6b,c shows that, with the decrease in PTFE content, the number of fibers at the crack of the reactive material decreased, and the diameter was also reduced to 0.1 μm. In reactive material No.4#, after adding CuO particles, the reinforced phase particles (CuO, Al) could still be well-capsulated by PTFE. However, only filamentous PTFE can be seen in reactive material No.7#, and the Al and Ni_2_O_3_ particles cannot be well capsulated, resulting in an excessive material gap.

Based on the above studies, it was found that the addition of CuO thermite to PTFE-based reactive materials helped improve the strength of the material after sintering, and the strength and content (less than 30 wt.%) changed in the same direction. The addition of Ni_2_O_3_ thermite into PTFE-based reactive materials can also improve the strength of the material after sintering; however, with the increase in the content, the strength first increases and then decreases because too many reinforcement particles destroy the continuity of the PTFE matrix. Therefore, increasing the content of CuO particles in PTFE/Al/CuO reactive materials will eventually lead to a decrease in strength.

### 3.3. Dynamic Impact Reaction Performance

**Figure 7:**
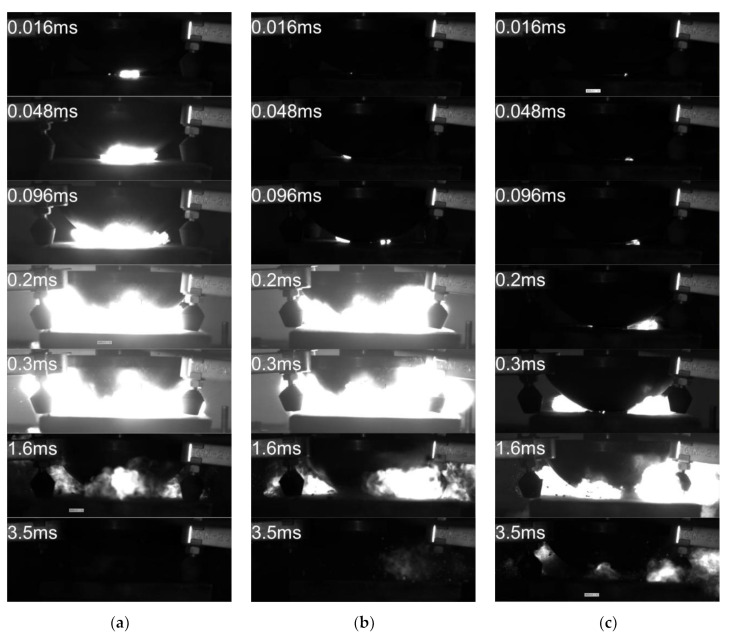
Typical pattern impact reaction process. (**a**) PTFE/Al; (**b**) PTFE/Al/CuO; (**c**) PTFE/Al/Ni_2_O_3_.

At a 25 °C ambient temperature and standard atmospheric pressure, a drop hammer test was carried out on No.1# through No.7# reactive materials. Each reactive material was tested 5 times, and the reaction process was photographed using a high-speed camera. Each experiment showed successful reactions. Because the reaction process was similar, we selected the impact reaction process of reactive materials No.1#, No.4#, and No.7#, as shown in Figure 7.

The reactive material first deformed under the impact of the drop hammer. After the material was destroyed, a violent reaction occurred between the materials, and a hot spot was formed inside the material, causing the material to emit light. The reaction was very rapid and emitted large amounts of light and heat.

**Figure 8:**
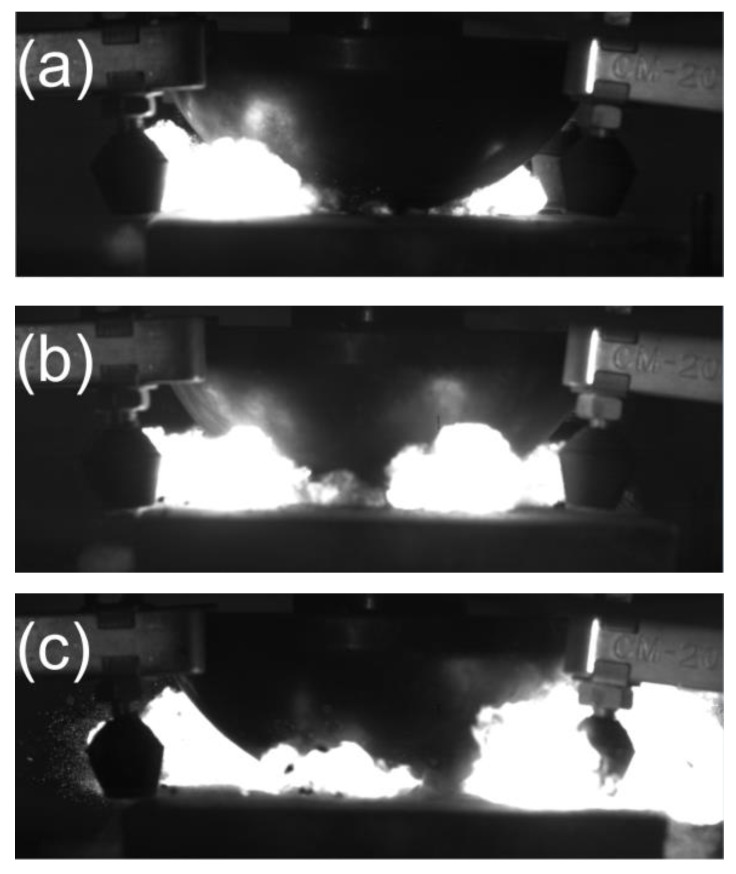
The most intense moments of reactions for different samples. (**a**) No.5# sample; (**b**) No.6# sample; (**c**) No.7# sample.

**Figure 9:**
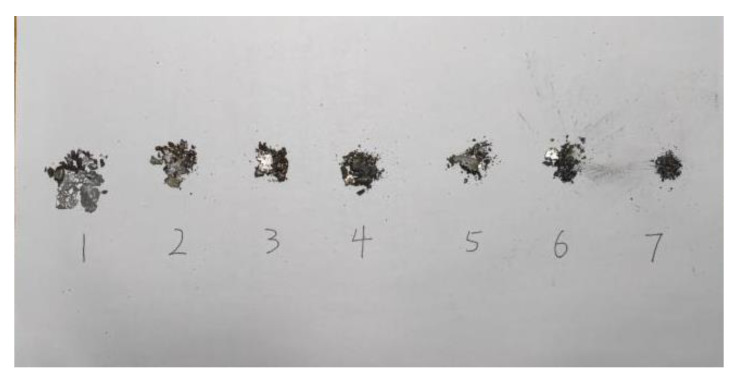
Residual material after the reactions.

Figure 8 shows the reaction durations of all samples. Since the energy release efficiency of the reactive materials under the drop hammer impact was less than 20 wt.% [24], the size of the fireball could reflect the energy release efficiency of the reactive material to a certain extent. Since the height of the fireball was obscured by the hammer, we designed its shape hemispherically to determine the size of the fireball more accurately by using the width range of the fireball. It can be seen from the diagram that, with the increase in Al/Ni_2_O_3_ content in the PTFE/Al/Ni_2_O_3_ reactive material, when the content was less than 20 wt.%, the size of the fireball was small and the change was not large. When the content was higher than 20 wt.%, the size of the fireball increased significantly. Based on the residual collected after the reaction, which is shown in Figure 9, it can be seen that, after the reaction, reactive material No.7# remained a fine powder material without a large amount of residue. The conclusion can be drawn that when the Al/Ni_2_O_3_ thermite content reaches 30 wt.%, the energy release efficiency of the reactive material is highest.

The duration of the four reactions in each group of the seven experiments was counted (excluding the secondary reaction time of reactive material No.7#). After removing the abnormal reaction duration, the average value was taken to obtain the reaction duration for each group of reactive materials, as shown in Table 3. It can be seen from the table that the reaction duration of the samples, with different formulations compacted at the same volume, was significantly different in the drop hammer system. The reaction duration of the reactive material with the Al/CuO thermite increased from 2 ms to 3.5 ms with the increase in the content. For the reactive materials with Al/Ni_2_O_3_ thermite, the reaction duration of the No.5# reactive material was 1.5 ms, and the reaction duration of the No.6# reactive material was 1.3 ms, all of which are less than the reaction duration of the PTFE/Al reactive materials. With the increase in content, the reaction duration increased suddenly. The reaction duration of the reactive material containing 30 wt.% Al/Ni_2_O_3_ thermite reached 4 ms.

Whether the abnormal reaction duration of the No.6# reactive material was caused by the insufficient height of the hammer was considered. The No.6# and No.7# reactive materials were tested again after the height was increased to 110 cm. It was found that the reaction duration of the No.6# reactive material was still 1.3 ms, while the reaction duration of the No.7# reactive material was slightly increased to 4.2 ms.

**Figure 10:**
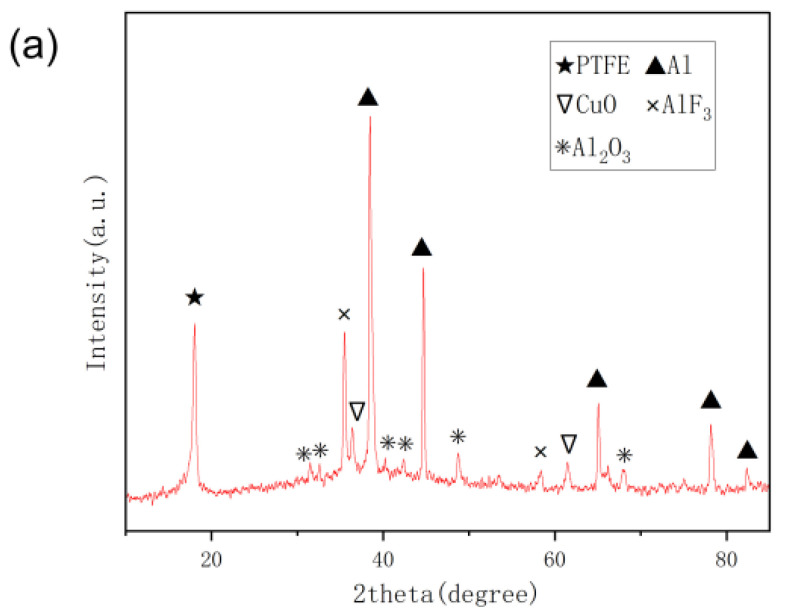

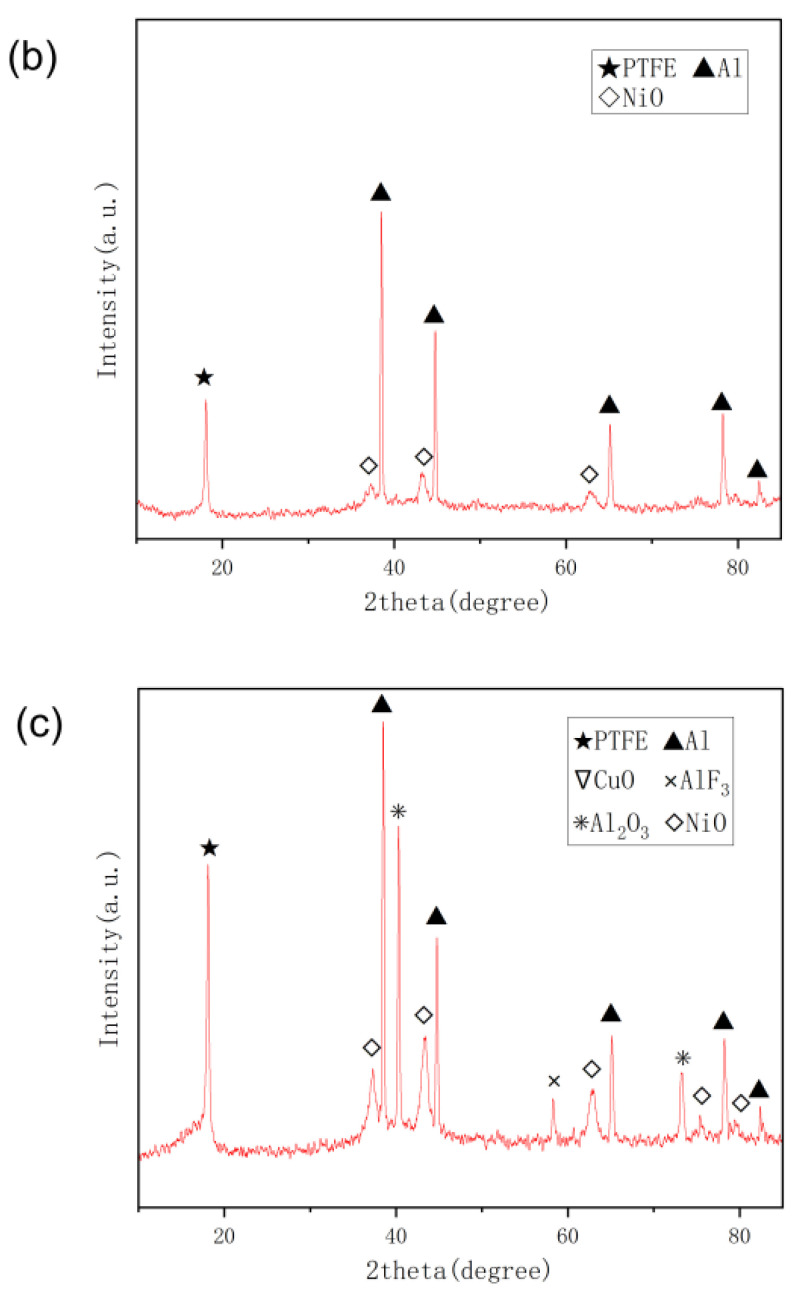
XRD analysis of different reactive material residues. (**a**) No.3# sample residue; (**b**) No.5# sample residue; (**c**) No.7# sample residue.

Subsequently, in order to further analyze the reasons, the reaction residues of the No.3# reactive material with the longest reaction duration, the No.5# reactive material with an abnormal reaction duration, and the No.7# reactive material with the longest reaction duration in the PTFE/Al/CuO reactive materials were selected for XRD analysis. The results are shown in Figure 10. From Figure 10a, it can be seen that the diffraction peaks of Al_2_O_3_ and AlF_3_ were detected in the residue of the No.3# reactive material. Combined with the residual black solid powder (which should be amorphous carbon black) after the reaction, it can be inferred that Al reacted with PTFE and CuO. Among them, Cu was difficult to collect due to the low content in the residue, and the XRD results did not show the presence of Cu. From Figure 10b, it can be seen that the diffraction peaks of Al_2_O_3_ and AlF_3_ were not detected in the residue of the No.5# reactive material, but the residual amorphous carbon black can be seen in the residue after the reaction. This indicates that the reaction of Al and PTFE occurred in the No.5# reactive material, but the reaction was not sufficient, so the content of AlF_3_ in the collected residue was not enough to be shown in the XRD results. The reaction of Al and Ni_2_O_3_ did not occur, which was confirmed by both the residue after the reaction and the XRD results. It can be seen from Figure 10c that the diffraction peaks of Al_2_O_3_, NiO, and AlF_3_ were detected in the residue of the No.7# reactive material, which indicated that Al reacted with PTFE and Ni_2_O_3_, and the reaction was sufficient.

As can be seen from Figure 10, the diffraction peaks of NiO were detected in the reaction residues No.5# and No.7#, but no diffraction peak of Ni_2_O_3_ was found because Ni_2_O_3_ reacted and decomposed into NiO and O_2_ above 600 °C:

2Ni_2_O_3_ = 4NiO + O_2_ (4)

This shows that part of Ni_2_O_3_ was reduced to NiO under the high-temperature conditions produced by the impact reaction of Al and PTFE, while the other part of Ni_2_O_3_ reacted with Al, and the generated nickel element quickly reacted with oxygen in the air to form NiO:

2Ni + O_2_ = 2NiO (5)

According to the XRD test results, it can be inferred that most of the NiO in reactive material No.5# was produced by the reduction of Ni_2_O_3_.

Under impact conditions, PTFE and Al first started to react at 625 °C [17], while Ni_2_O_3_ decomposed into NiO and O_2_ over 600 °C. However, the excitation temperature of the thermite reaction was between 1000 °C and 1400 °C. When the content of Al/Ni_2_O_3_ thermite was less than 20%—because the content of Ni_2_O_3_ was small (under the heat released from the reaction of PTFE and Al)—the internal heat reached the decomposition condition of Ni_2_O_3_ first, and most of Ni_2_O_3_ was decomposed rapidly before it reacted with Al. At the same time, the generated oxygen made the gap of the specimen bigger, which affected the further contact reaction of the PTFE and Al particles, leading to the reaction stopping quickly. The size of the fireball at the strongest reaction time of the No.5# and No.6# reactive materials was smaller than that of the No.1# reactive materials, and the reaction duration is also low. When the content of Al/Ni_2_O_3_ thermite was high, the heat continued to increase with the reaction of PTFE and Al, and part of the Ni_2_O_3_ still decomposed. After the heat reached the condition of the thermite reaction, the Ni_2_O_3_ particles were not fully decomposed and the reaction of Al and Ni_2_O_3_ could occur, releasing a large amount of heat and resulting in the longest reaction duration and the largest fireball size of reactive material No.7#.

## 4. Conclusions

Through comparative experimental studies of PTFE/Al/Ni_2_O_3_ reactive materials, the following conclusions were obtained:

1. The addition of oxides can improve the mechanical properties of PTFE/Al reactive materials to a certain extent, but the addition of different oxides (CuO and Ni_2_O_3_) results in little difference in the mechanical properties. With the increase in Ni_2_O_3_ content, the continuity of the matrix would be destroyed, the strength of the reactive material would be reduced, and the material would soon fail after the elastic stage.
2. During the dynamic impact test, seven groups of reactive materials with different ratios can have an impact explosion reaction. When the content of Al/Ni_2_O_3_ added to PTFE/Al was 30%, the reaction was the most intense, and the reaction duration was 21% higher than when adding the same content of Al/CuO. Through XRD characterization, it was found that the PTFE/Al/Ni_2_O_3_ reactive material produced NiO in two ways during the reaction process. Further, when the content of Al/Ni_2_O_3_ was less than 20 wt.%, the thermite reaction was more difficult to achieve, resulting in a shorter reaction time and lower intensity.

## Figures and Tables

**Table 1:** Material number and ratio.

No.	(73.5%) PTFE + (26.5%) Al (µm) (wt.%)	(81.5%) CuO + (18.5%) Al (nm) (wt.%)	(75.4%) Ni_2_O_3_ + (24.6%) Al (nm) (wt.%)
1#	100	-	-
2#	90	10	-
3#	80	20	-
4#	70	30	-
5#	90	-	10
6#	80	-	20
7#	70	-	30

**Table 2:** Volume fraction of oxides in reactive materials.

Material No.	1#	2#	3#	4#	5#	6#	7#
Oxide vol%	0	1.29	2.58	3.87	1.56	3.12	4.69

**Table 3:** Reaction duration of reactive materials.

Material No.	1#	2#	3#	4#	5#	6#	7#
Reaction duration (ms)	2.6	2	2.3	3.5	1.5	1.3	4.0

## Data Availability

The data presented in this study are available upon request from the corresponding author.
